# Rétraction - double publication de l´article: Prise en charge anesthésique des cardiopathies congénitales opérées sous circulation extra - corporelle au Centre de Chirurgie Cardiaque Pédiatrique Cuomo (Sénégal) par Farid Pingwindé Belem et al. (doi: 10.11604/pamj.2020.37.362.17659)

**DOI:** 10.11604/pamj.2021.38.177.28413

**Published:** 2021-02-16

**Authors:** Pan African Medical Journal

**Affiliations:** 1 The Pan African Medical Journal, Yaoundé, Cameroun

**Keywords:** Double publication, retraction, PAMJ, Double publication, Retraction, PAMJ

## Abstract

Cet article rétracte l´article “Prise en charge anesthésique des cardiopathies congénitales opérées sous circulation extra - corporelle au Centre de Chirurgie Cardiaque Pédiatrique Cuomo (Sénégal)”. Farid Pingwindé Belem, El Hadji Boubacar Ba, Papa Alassane Leye, El Hadji Ndiassé Diop, Ibrahima Gaye, Mamadou Diawo Bah, Etienne Birame Sène, Marie Victoire Sène, Mohamed Lamine Fall, Amadou Gabriel Ciss, Oumar Kane. The Pan African Medical Journal. 2020;37:3362. (doi:10.11604/pamj.2020.37.362.17659)”.

## Abstract (en)

This article retracts article “Anaesthesia in on-pump cardiac surgery for congenital heart diseases at the Cuomo Pediatric Cardiology Center (Senegal)”. Farid Pingwindé Belem, El Hadji Boubacar Ba, Papa Alassane Leye, El Hadji Ndiassé Diop, Ibrahima Gaye, Mamadou Diawo Bah, Etienne Birame Sène, Marie Victoire Sène, Mohamed Lamine Fall, Amadou Gabriel Ciss, Oumar Kane. The Pan African Medical Journal. 2020;37:3362. (doi:10.11604/pamj.2020.37.362.17659)”

## Rétraction

Nous tenons à notifier les lecteurs de PAMJ de la rétraction de l´article *Prise en charge anesthésique des cardiopathies congénitales opérées sous circulation extra - corporelle au Centre de Chirurgie Cardiaque Pédiatrique Cuomo (Sénégal)* (doi: 10.11604/pamj.2020.37.362.17659). Farid Pingwindé Belem, El Hadji Boubacar Ba, Mamadou Diawo Bah, Etienne Birame Sène, Marie Victoire Sène, Mohamed Lamine Fall, Oumar Kane du Service d'Anesthésie - Réanimation, CHU de Fann, Dakar, Sénégal, Papa Alassane Leye, Ibrahima Gaye, El Hadji Ndiassé Diop du Service d'Anesthésie - Réanimation, CHU Aristide Ledantec, Dakar, Sénégal et Amadou Gabriel Ciss du Service de Chirurgie Thoracique et Cardiovasculaire, CHU de Fann, Dakar, Sénégal [1]. Au terme de notre investigation, il apparait de manière indiscutable que le manuscrit soumis par Farid Pingwindé Belem *et al*. et publié dans notre journal le 21 décembre 2020, avait été publié précédement dans un autre journal par les auteurs. En effet, le manuscrit soumis par Farid Pingwindé Belem, El Hadji Boubacar Ba, Papa Alassane Leye, El Hadji Ndiassé Diop, Ibrahima Gaye, Mamadou Diawo Bah, Etienne Birame Sène, Marie Victoire Sène, Mohamed Lamine Fall, Amadou Gabriel Ciss, Oumar Kane et publié dans PAMJ est une copie conforme du manuscrit *Prise en charge anesthésique des cardiopathies congénitales opérées sous circulation extra-corporelle au centre de chirurgie cardiaque pédiatrique* publié dans Médecine d'Afrique Noire 6702 - Février 2020 - pages 80-90 [2] par M.L. Fall, P.F. Belem, P.A. Leye, E.H. Ba, E.H.N. Diop, I. Gaye, M.D. Bah, E.B. Sene, M.V Sene, O. Kane.

Par conséquent, nous retirons cet article de la littérature médicale en conformité avec les lignes directrices et les meilleures pratiques éditoriales du *Committee on Publication Ethics*. Nous nous excusons auprès de notre public pour cette situation malheureuse.
